# An Open-Source Graphical User Interface-Embedded Automated Electrocardiogram Quality Assessment: A Balanced Class Representation Approach

**DOI:** 10.3390/diagnostics13223479

**Published:** 2023-11-20

**Authors:** Mohamed Elgendi, Kirina van der Bijl, Carlo Menon

**Affiliations:** Biomedical and Mobile Health Technology Lab, Department of Health Sciences and Technology, ETH Zurich, 8008 Zurich, Switzerland

**Keywords:** electrocardiograms (ECGs), digital medicine, artificial intelligence, digital health, CNN-LSTM model, user-friendly toolbox

## Abstract

The rise in cardiovascular diseases necessitates accurate electrocardiogram (ECG) diagnostics, making high-quality ECG recordings essential. Our CNN-LSTM model, embedded in an open-access GUI and trained on balanced datasets collected in clinical settings, excels in automating ECG quality assessment. When tested across three datasets featuring varying ratios of acceptable to unacceptable ECG signals, it achieved an F1 score ranging from 95.87% to 98.40%. Training the model on real noise sources significantly enhances its applicability in real-life scenarios, compared to simulations. Integrated into a user-friendly toolbox, the model offers practical utility in clinical environments. Furthermore, our study underscores the importance of balanced class representation during training and testing phases. We observed a notable F1 score change from 98.09% to 95.87% when the class ratio shifted from 85:15 to 50:50 in the same testing dataset with equal representation. This finding is crucial for future ECG quality assessment research, highlighting the impact of class distribution on the reliability of model training outcomes.

## 1. Introduction

Globally, cardiovascular diseases (CVDs) are the leading cause of death, accounting for 32% of all global fatalities in 2019, with the majority occurring in low- to middle-income countries [1]. Heart attacks and strokes, which comprise 85% of these CVD-related deaths, underscore the urgent need for effective diagnostic tools, such as Electrocardiography (ECG) [2,3,4]. The advent of wearable technologies has facilitated continuous ECG monitoring, significantly advancing cardiovascular care [5,6]. However, the interpretation of ECG records is complicated due to their vulnerability to disruptions [7].

Advancements in ECG integration with digital health technologies offer promising solutions to improve cardiac care, particularly in underserved regions [8,9]. Automated ECG quality assessment is pivotal in these settings, where access to healthcare and trained specialists is limited. By enabling the immediate identification and re-recording of subpar ECGs, these techniques can streamline the diagnostic process, reducing delays and improving patient outcomes [10,11].

The traditional approach to ECG recording and assessment, often manual and time-consuming, demands expertise [12,13]. Modern approaches leveraging machine learning and sensor advancements offer a more efficient alternative, potentially reducing the workload on healthcare professionals [14,15,16]. Moreover, real-time ECG quality assessment techniques, incorporating advanced signal processing, play a crucial role in the reliability and accuracy of diagnoses [17,18].

Our recent works have demonstrated the versatility of ECG in various contexts, from assessing driving stress to hypertension monitoring, further highlighting its potential in cardiovascular care [19,20]. The democratization of ECG assessments through automated systems can make them more accessible and practical in diverse healthcare environments [21,22].

Building on these motivations, our study introduces a new machine learning algorithm embedded in a graphical user interface (GUI), designed to enhance usability for clinicians. This tool not only eases the assessment of ECG signal quality, but also considers class representation during training and testing phases, addressing a crucial aspect often overlooked in previous studies.

## 2. Methodology

### 2.1. Training Datasets: CinC11 and CinC17

The CinC11 dataset [23] used in this study was a relabeled single-lead version. Each lead was assigned a separate label, unlike the original dataset with 12-leads having a single label. The relabeled dataset, available on GitHub [24], consisted of 5400 10-s single-lead ECG recordings from CinC11. These recordings were divided into a train set of 1200 samples and a test set of 4200 samples. The train set comprised 67.92% acceptable signals and 32.08% unacceptable signals. A random split was performed, creating a train-validation ratio of 90:10. The test set contained 67.71% acceptable signals and 32.29% unacceptable signals, as defined in [24].

For training an alternative version of the CNN-LSTM model, a mixture of CinC11 [23] and CinC17 [25] data were used. One dataset aimed for a balanced distribution of acceptable and unacceptable labeling with a 50:50 ratio. Additional noisy data from CinC17 was incorporated to balance the classes and generate more training data. This resulted in a dataset of 4048 10-s ECG recordings, with 2024 samples labeled as acceptable and 2024 samples labeled as unacceptable.

The second dataset included all data from both CinC11 and CinC17, resulting in a larger dataset of 13,912 samples. Out of these, 11,888 were labeled as acceptable and 2024 samples were labeled as unacceptable. The larger dataset size aimed to enhance the model’s flexibility and robustness due to the availability of more training data.

Both datasets were split into training, validation, and test sets using an 80:10:10 ratio. However, it is important to note that the randomization algorithm used for splitting the data makes it difficult to trace the origin of leads within the splits. It is possible that leads from a single 12-lead recording ended up in different sets, which could potentially introduce data leakage and inflate the results [26,27].

### 2.2. Testing Dataset: BUT QDB

The BUT QDB dataset [28,29] was used as an external test dataset to avoid high performance scores due to leakage. The original 20 min recordings were preprocessed to consist of multiple 10 s samples with a single label. All the labeled segments shorter than 10 s were discarded. This resulted in 10,570 samples. The BUT QDB dataset consists of 1000 Hz recordings, so the 10,570 samples of 10 s were downsampled to become 500 Hz. A total of 5285 of these samples were labeled *acceptable,* and 5285 samples were labeled *unacceptable*.

### 2.3. Spectrogram Conversion

ECG recordings were first normalized between −1.0 and 1.0 before being converted into spectrograms using the *tf.signal.stft* function. This function applies to the ECG tensor of shape (samples, channels), with channels being *None*, as the ECG tensor comprises a single channel. Key parameters for the function are frame length and frame step, denoting the length and step size of the analysis window, respectively, used in the Short-Time Fourier Transform (STFT) computation.

The STFT process splits the ECG tensor into frames, applying the Fourier transform separately to each, thus switching the signal from the time domain to the frequency domain. The window function employed here, the Hann function, aids in mitigating spectral leakage, or the distortion of signal frequency when it extends beyond the window edges. The selection of the frame length is determined by a trade-off between time and frequency resolution.

STFT is given by:

(1) $$STFTx[k]=X\left(m,\omega \right)=\sum_{\infty }^{k=-\infty }x\left[k\right]\omega \left[k-m\right]e^{-j\omega k},$$

where *k* indicates the index of the analyzed value in the ECG data sample, and the fast Fourier transform values are computed over discrete steps or ‘windows’.

The Hann window function, used here, is defined as:

(2) $$w\left[n\right]=0.5-0.5\cos\left(2\pi \frac{n}{N}\right).$$

This function’s bell-shaped and symmetric nature transitions from 0 at the window start and end to 1 in the center, reducing spectral leakage. Chosen over the Hamming window function due to its superior frequency-time resolution trade-off and effectiveness in reducing spectral leakage, the Hann window function is more suitable for ECG data, particularly for identifying high-frequency arrhythmias such as atrial fibrillation.

### 2.4. CNN-LSTM Model

The CNN-LSTM model used in this study is based on the work of Özer et al. [30], originally designed for classifying power quality disturbances in the power grid. The model consists of two main parts: a Convolutional Neural Network (CNN) for feature extraction and a Long Short-Term Memory (LSTM) network for temporal data analysis and classification. The input data, originally power grid data, is converted into two-dimensional spectrograms before being fed into the model. The CNN part performs feature extraction, while the LSTM part captures temporal dependencies and performs classification. However, to adapt the model for ECG quality assessment, several modifications were made to reduce overfitting and computational demands. Specifically, additional dropout layers were added to the CNN part, and the Bi-LSTM was replaced with an LSTM. This modified model closely resembles models used in previous studies for heartbeat event classification and atrial fibrillation detection.

**Figure 1:**
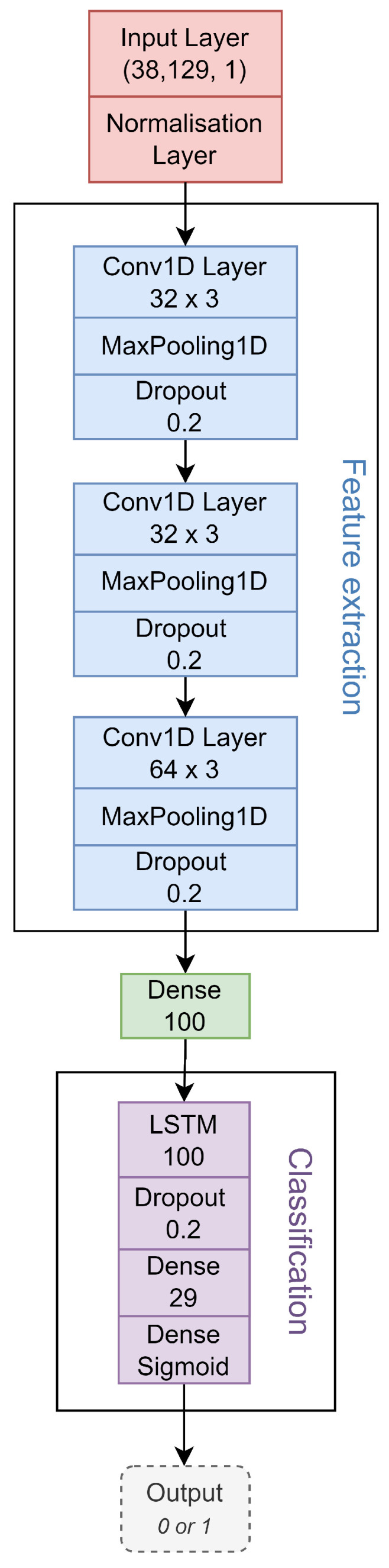
Schematic overview of the proposed CNN-LSTM network. The *Feature extraction* box outlines the CNN part of the model. The *Classification* box outlines the LSTM part of the model. The values under the input layer represent the input shape. The values under the Conv1D layers represent the filter and kernel size. The value under the dropout layers represents the dropout rate. The value under the LSTM and Dense layers represents the dimensionality of the output. The Sigmoid under the last Dense layer represents the activation function of the output.

The finalized CNN-LSTM model, illustrated in Figure 1, starts with an input layer that takes in spectrogram data of shape (38, 129). A normalization layer is applied to scale the input data for improved processing. The model then incorporates a series of convolutional layers, max pooling layers for down-sampling, dropout layers to prevent overfitting, and dense layers for feature extraction. The LSTM layer, a type of recurrent neural network, follows the CNN part and captures temporal dependencies in the sequential data. After additional dropout and dense layers, a sigmoid activation function is applied in the output layer to predict the class probabilities for ECG quality assessment.

For model training, we utilized the Adam optimizer, an optimization algorithm favored for its adaptive learning rate, which adjusts during training based on parameter gradients. This contrasts with algorithms like stochastic gradient descent, which employ a static learning rate throughout training.

Training the model involved the Adam optimizer with a learning rate of 0.0001 and sparse categorical cross-entropy loss. The learning rate parameter impacts the model parameters’ modification speed with each iteration. Lower rates make for slower, more accurate updates, while higher rates expedite these updates, but may reduce precision. Here, we have opted for a 0.0001 learning rate as the typical 0.001 rate seemed to hasten model convergence excessively. Despite altering the training ‘friction’ level, this learning rate does not affect the adaptive nature of the Adam optimizer.

Following training, we saved the parameters for future testing and integration into the toolbox. We carried out the implementation using Python 3.8.11 and TensorFlow 2.9.1, leveraging both a CPU and GPU for training. For a dataset of 13,912 data points, the training process lasted around 10 min.

## 3. Results

**Figure 2:**
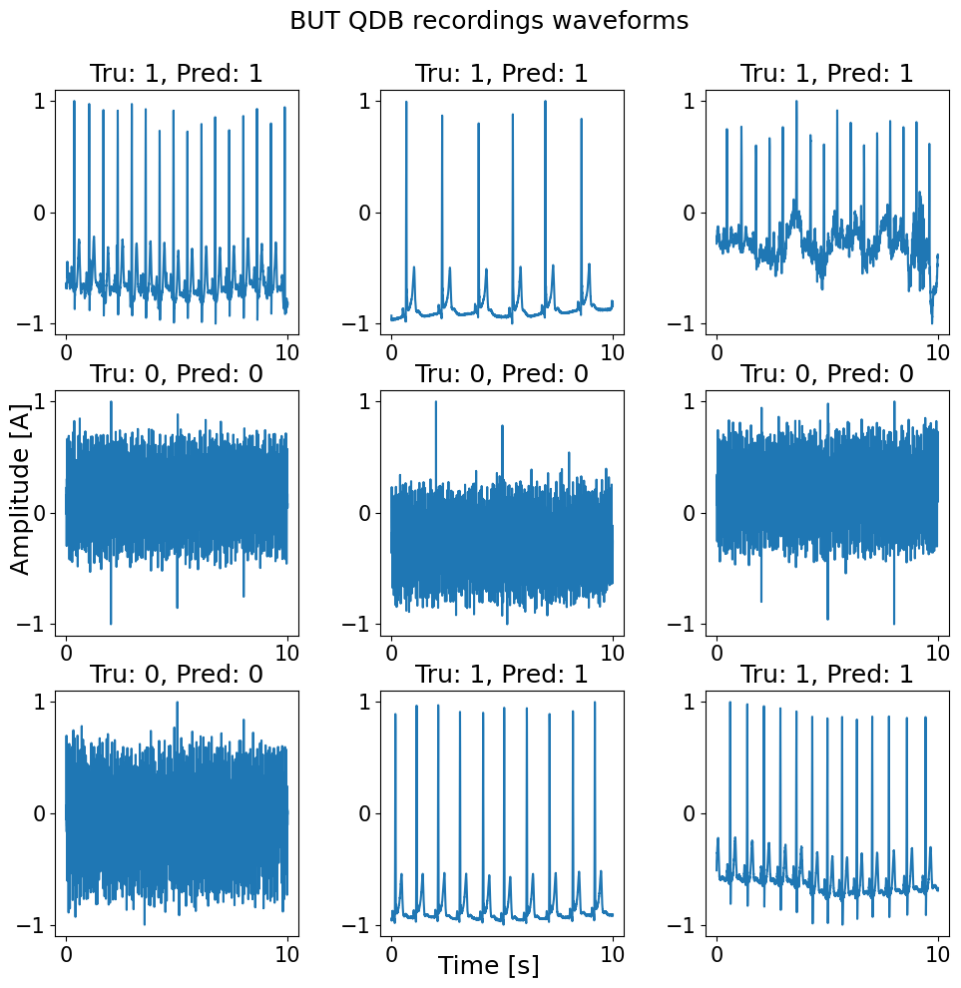
Examples of true positives and true negatives yielded by the proposed CNN-LSTM model, utilizing the BUT QDB dataset.

Figure 2 shows the results of the CNN-LSTM model on waveform input data, the figure displays the ground truth labels [‘Tru’] and the predicted labels [‘Pred’].

Table 1 summarizes the performance of the proposed CNN-LSTM model compared to other methods. The model achieved the highest sensitivity, specificity, accuracy, and F1-score among all the analyzed methods.

The toolbox underwent significant changes to improve usability. It now accepts waveform data without a specific numbering scheme, can import .wav files, automatically detects the number of leads in the data, and adjusts the table size accordingly. The code was also optimized and follows software engineering best practices.

**Figure 3:**
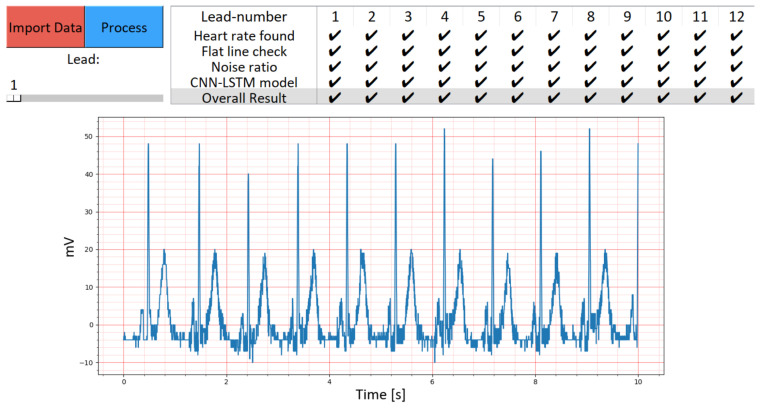
Showcase of the new toolbox with an instance of an ECG signal with acceptable quality. The GUI marks it as bad quality due to automatic labeling based on the proposed CNN-LSTM algorithm. Other ECG signal quality indices in agreement. The displayed ECG corresponds to ‘Lead 1’, extracted from the CinC11 dataset, which includes 12-lead ECG data. By utilizing the scroll bar situated in the top left corner of the graphical user interface, we can load and examine each ECG lead within the dataset. Remarkably, all 12 ECG leads exhibit acceptable quality.

**Figure 4:**
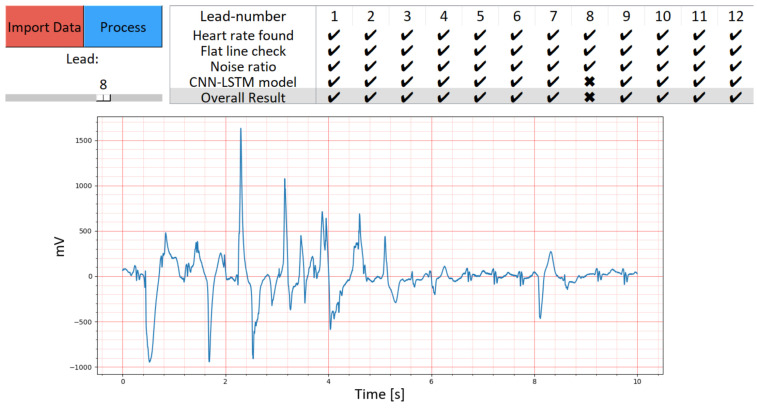
Showcase of the new toolbox with an instance of an ECG signal with unacceptable quality. The GUI marks it as bad quality due to automatic labeling based on the proposed CNN-LSTM algorithm. Other ECG signal quality indices in disagreement. The displayed ECG corresponds to ‘Lead 8,’ extracted from the CinC11 dataset, which includes 12-lead ECG data. By utilizing the scroll bar situated in the top left corner of the graphical user interface, we can load and examine each ECG lead within the dataset. Remarkably, all 12 ECG leads, excluding ‘Lead 8,’ exhibit acceptable quality.

The new toolbox is shown in Figure 3 and Figure 4, and the code for the toolbox can be found on GitHub (https://github.com/Kirina/Automated_ecg_assessment, accessed on 16 November 2023), along with detailed instructions on its usage. The original code is also available on GitHub (https://github.com/LinusKra/ECGAssess, accessed on 16 November 2023), enabling easy comparison of the code changes. Figure 3 presents a demonstration highlighting the CNN-LSTM’s capability to automatically classify ‘Lead 1’ as having acceptable quality. In contrast, Figure 4 illustrates an instance of unacceptable quality in a distinct ECG recording.

## 4. Discussion

Our evaluation of the CNN-LSTM model across diverse datasets such as CinC11 [23], CinC17 [25], and BUT QDB [28,29] demonstrates its adaptability and robustness. The model’s effective performance on the BUT QDB dataset, which was gathered under different conditions than the training data, underscores its generalizability.

In terms of sensitivity, specificity, accuracy, and F1-score, the CNN-LSTM model exhibits superior effectiveness in ECG quality assessment, outperforming other methods as detailed in Table 1. This highlights its potential for practical clinical application. Moreover, our analysis showed the performance of the proposed algorithm with equal representations of high quality vs. low quality, at 50:50, during both the training and testing phases. This highlights the importance of class representation during training and testing. Notably, the F1 score of the proposed algorithm decreased from 98.09% to 95.87% when the class representation changed from 85:15 to 50:50, despite the test set having an equal representation for both. This point must be carefully considered when reporting results in future investigations on this topic.

In our study, the newly developed toolbox was demonstrated using examples from the CinC11 dataset, as illustrated in Figure 3. This figure showcases the toolbox’s interface while analyzing an ECG signal from ‘Lead 1’, marked as having acceptable quality. Despite the CNN-LSTM algorithm’s automatic labeling indicating bad quality, other ECG signal quality indices concurred with the acceptable quality assessment. This instance demonstrates the toolbox’s ability to facilitate a comprehensive review of ECG signals. The user-friendly graphical interface, complete with a scroll bar, allows clinicians to easily navigate and assess each of the 12 leads within an ECG recording. The effective visualization and assessment capabilities of the toolbox, as seen in Figure 3, underscore its potential utility in clinical settings.

Figure 4 provides an additional perspective on the toolbox’s functionality by presenting an ECG signal from ‘Lead 8’ of the CinC11 dataset, this time marked as having unacceptable quality. In this case, the GUI’s labeling based on the CNN-LSTM algorithm indicates bad quality, aligning with the disagreement observed in other ECG signal quality indices. This example highlights the toolbox’s sensitivity in detecting and labeling poor-quality signals, an essential feature for ensuring accurate ECG analysis. The graphical user interface again proves instrumental in allowing for the detailed examination of each lead, reinforcing the toolbox’s role in enhancing ECG quality assessment. The contrast between the results for ‘Lead 1’ and ‘Lead 8’ within the same dataset illustrates the model’s nuanced approach to ECG signal evaluation.

A primary strength of this study is the extensive evaluation of the model across varied datasets, which reduces the risk of dataset-specific overfitting and showcases the model’s ability to handle different signal qualities. Combining CNN for feature extraction with LSTM for temporal data analysis, the model adeptly identifies complex ECG patterns, resonating with the guidelines in [36]. This methodology significantly contributes to discussions on the need for robust and adaptable models in biomedical signal analysis, particularly for diagnosing noncommunicable diseases.

Furthermore, the study emphasizes transparency and reproducibility, demonstrated by the public availability of the source code [24]. This openness encourages further research and collaboration in the field.

However, the study has limitations. The model’s effectiveness is closely linked to the diversity and quality of the training data, and its performance might be impacted when exposed to highly varied data types. The computational demands for training and deploying deep learning models could also limit their feasibility in certain settings.

Future research should focus on developing algorithms that are compatible with various ECG recording devices by accommodating multiple sampling frequencies. Expanding the algorithm to handle different data lengths and enabling real-time analysis could greatly enhance its clinical utility. Exploring hybrid models that combine feature-based and deep learning approaches might offer a balance between computational efficiency and interpretability.

The consistent and versatile performance of our CNN-LSTM model across various datasets reaffirms its promising reliability as a screening tool. This tool has the potential to improve decision-making processes when collecting ECGs in different settings with noise and various clinical environments. Feedback from clinicians on this tool would be highly appreciated to understand its efficacy, scalability, and effectiveness in improving cardiac diagnosis and treatment.

## 5. Conclusions

This study introduces an innovative CNN-LSTM model for automated ECG quality assessment, embedded in an open-access GUI and trained on real-world noise without simulated data. The model, comprising a CNN for feature extraction and an LSTM for classification, shows superior performance on various datasets, including CinC11, CinC17, and BUT QDB. It demonstrates high sensitivity, specificity, accuracy, and F1 scores, highlighting its robustness in diverse clinical settings. The balanced class representation during training provides crucial insights, emphasizing the need for accurate class distribution in ECG quality assessment. Future work should focus on enhancing the model’s adaptability to different data types and extending its capabilities for real-time analysis.

## Figures and Tables

**Table 1:** Overview of the performance of the proposed CNN-LSTM compared to other methods. Ac refers to acceptable, UnAc refers to unacceptable. N/R refers to not received, meaning that the value was not mentioned in the article. ^1^ Subset of CinC11 labeled in [24]. ^2–6^ Same dataset. ^7^ Calculated value from values in paper.

Method	Year	Train Dataset	Test Dataset	Train Ratio	Test Data	Sensitivity	Specificity	Accuracy	F1-Score
				**Ac:UnAc**	**Ac:UnAc**	**(%)**	**(%)**	**(%)**	**(%)**
Proposed method	2022	CinC11 ^1^ and CinC17 ^2^	BUT QDB ^3^	50:50	50:50	92.43	99.60	96.02	95.87
Proposed method	2022	CinC11 ^1^ and CinC17 ^2^	CinC11 ^1^ and CinC17	50:50	50:50	98.52	95.52	97.03	97.09
Proposed method	2022	CinC11 ^1^ and CinC17 ^4^	BUT QDB ^3^	85:15	50:50	99.41	96.71	98.06	98.09
Proposed method	2022	CinC11 ^1^ and CinC17 ^4^	CinC11 ^1^ and CinC17	85:15	85:15	99.74	83.80	97.27	98.40
Proposed method	2022	CinC11 ^1,5^	CinC11 ^1,6^	68:32	68:32	98.24	92.04	96.23	97.25
Kramer et al. [24]	2022	CinC11 ^1,5^	CinC11 ^1,6^	68:32	68:32	98.03	86.21	94.21	96.31 ^7^
Hermawan et al. [31]	2019	CinC11	CinC11	70:30	70:30	85.00	86.00	85.60	N/R
Clifford et al. [32]	2012	CinC11 and NSTDB	CinC11 and NSTDB	50:50	50:50	N/R	N/R	95.80	N/R
Taji et al. [33]	2017	CinC11 and NSTDB	CinC11 and NSTDB	50:50	50:50	98.20	98.20	97.20	98.38
Yaghmaie et al. [34]	2017	CinC11 and NSTDB and	CinC11 and NSTDB	50:50	50:50	96.20	97.60	96.90	N/R
		MIT-BIH							
Fu et al. [35]	2021	Private	Private	80:20	84:16	98.66	86.65	96.73	N/R

## Data Availability

The CinC11, CinC17, and BUT QDB datasets can be downloaded via the following links: CinC11: https://physionet.org/content/challenge-2011/1.0.0/, accessed on 16 November 2023; CinC17: https://archive.physionet.org/pn3/challenge/2017/, accessed on 16 November 2023; BUT QDB: https://physionet.org/content/butqdb/1.0.0/, accessed on 16 November 2023. The associated code is publicly accessible and can be downloaded from https://github.com/Kirina/Automated_ecg_assessment, accessed on 16 November 2023.
